# Inflammatory Indexes for Assessing the Severity and Disease Progression of Ulcerative Colitis: A Single-Center Retrospective Study

**DOI:** 10.3389/fpubh.2022.851295

**Published:** 2022-03-10

**Authors:** Hanyang Lin, Zhaohui Bai, Qiong Wu, Guiyang Chu, Yongguo Zhang, Xiaozhong Guo, Xingshun Qi

**Affiliations:** ^1^Department of Gastroenterology, General Hospital of Northern Theater Command, Shenyang, China; ^2^Postgraduate College, China Medical University, Shenyang, China; ^3^Postgraduate College, Shenyang Pharmaceutical University, Shenyang, China; ^4^Department of Thoracic Surgery, General Hospital of Northern Theater Command, Shenyang, China; ^5^Information Section of Medical Security Center, General Hospital of Northern Theater Command, Shenyang, China

**Keywords:** ulcerative colitis, inflammatory indexes, activity, severity, 5-aminosalicylic acid

## Abstract

**Background:**

Active and severe ulcerative colitis (UC) and non-response to 5-aminosalicylic acid (5-ASA) are related to poor outcomes and should be accurately identified. Several integrated inflammatory indexes are potentially useful to assess the disease severity in patients with acute or critical diseases but are underexplored in patients with UC.

**Methods:**

Patients with UC consecutively admitted to our hospital between January 2015 and December 2020 were retrospectively grouped according to the activity and severity of UC and response to 5-ASA. The neutrophil-to-lymphocyte ratio (NLR), platelet-to-lymphocyte ratio (PLR), systemic immune-inflammation index (SII), neutrophil-to-platelet ratio (NPR), platelet-to-albumin ratio (PAR), C-reactive protein-to-albumin ratio (CAR), and C-reactive protein-to-lymphocyte ratio (CLR) were calculated. The areas under receiver operating characteristic curves (AUC) were calculated.

**Results:**

Overall, 187 patients with UC were included, of whom 151 were active, 55 were severe, and 14 were unresponsive to 5-ASA. The active UC group had significantly higher NLR, PLR, SII, and PAR levels. SII had the greatest predictive accuracy for active UC, followed by PLR, PAR, and NLR (AUC = 0.647, 0.641, 0.634, and 0.626). The severe UC group had significantly higher NLR, PLR, SII, PAR, CAR, and CLR levels. CLR had the greatest predictive accuracy for severe UC, followed by CAR, PLR, SII, NLR, and PAR (AUC = 0.732, 0.714, 0.693, 0.669, 0.646, and 0.63). The non-response to the 5-ASA group had significantly higher CAR and CLR levels. CAR had a greater predictive accuracy for non-response to 5-ASA than CLR (AUC = 0.781 and 0.759).

**Conclusion:**

SII, CLR, and CAR may be useful for assessing the severity and progression of UC, but remain not optimal.

## Introduction

Ulcerative colitis (UC) is a relapsing and remitting mucosal inflammation often restricted to the colon and rectum, which may be associated with dysregulated immune response (1). The highest incidence and prevalence of UC is 4.6 per 100,000 person-years and 57.3 per 100,000 persons in Eastern Asia, respectively (2). Patients with active UC always suffer from embarrassing and painful symptoms, such as fecal incontinence, abdominal pain, bloody diarrhea, arthritis, and fatigue, and tend to develop poorer psychosocial outcomes than those with inactive UC (3). Active UC is usually classified into mild, moderate, and severe according to the recommendation by the international guideline (4). Generally, severe UC can bring more negative impact on the patient's quality of life, social and psychological wellbeing, healthcare resource utilization (5), and prognosis than mild-moderate UC (6, 7). Early medications can avoid the progression from mild-moderate to severe UC (7). 5-aminosalicylic acid (5-ASA) is the first-line choice of medication for patients with UC diagnosed within the first year (8). Usually, patients with severe UC are not well responsive to 5-ASA, leading to the use of corticosteroids, immunosuppressants, and biologics (9–12). Therefore, it is a clinical priority to identify patients who require more aggressive treatment to reach clinical remission.

In 1955, Truelove and Witts (13) established their criteria to explore the efficacy of cortisone medications in patients with UC. Currently, the Truelove and Witts criteria have been the cornerstone of assessing the severity of UC. However, it still has several potential limitations. The most critical limitation is that the definitions of improvement and worsening are ambiguous (14), as well as that of moderate UC. In 1987, Schroeder et al. (15) further developed the Mayo score by combining clinical symptoms, laboratory tests, and endoscopic findings. Despite one of the most commonly used scores for the severity of UC, it contains endoscopic procedures, which may be invasive, expensive, and time-consuming. Recently, the partial Mayo score (PMS) has been increasingly recognized, because it can properly discriminate this disease based on stool frequency, rectal bleeding, and physician's global assessment, but does not contemplate endoscopic data (16–18). However, the requirement of clinician's subjective evaluation of patient's symptoms remains its potential drawback (19).

Erythrocyte sedimentation rate (ESR) and C reactive protein (CRP) are two traditional serologic biomarkers and are usually used for monitoring the disease course of UC, but their sensitivity and specificity are unsatisfactory (20). Recently, several integrated indexes have been used for the assessment of infective, acute, and critical diseases, including neutrophil-to-lymphocyte ratio (NLR) (21), platelet-to-lymphocyte ratio (PLR) (22), systemic immune-inflammation index (SII) (23), neutrophil-to-platelet ratio (NPR) (24), platelet-to-albumin ratio (PAR) (25), CRP-to-albumin ratio (CAR) (26), and CRP-to-lymphocyte ratio (CLR) (27). Notably, they are non-invasive and easier to use at a low cost. Thus, the purpose of this study is to determine the accuracy of inflammatory indexes for diagnosing active UC and severe UC and identifying the response to 5-ASA medication.

## Methods

### Patient Selection

This is a single-center retrospective, cross-sectional study. We extracted the medical records of all UC patients who were consecutively admitted to the General Hospital of Northern Theater Command between January 1, 2015 and December 31, 2020 from the inpatient information system. The exclusion criteria were as follows: (1) medical records cannot be reviewed in detail; (2) patients were diagnosed as suspected UC and unclassified inflammatory bowel disease; (3) routine blood tests were missing; (4) history of colectomy; (5) co-existing conditions that potentially influence inflammatory indexes (i.e., severe trauma, pregnancy, liver cirrhosis, uremia, and malignancy); and (6) other autoimmune diseases (i.e., psoriasis, Behcet's disease, urticarial vasculitis, and rheumatoid arthritis). The study protocol was approved by the Ethical Committee of General Hospital of Northern Theater Command [Y (2021) 078] and conformed to the ethical guidelines of the 1975 Declaration of Helsinki. The requirement of informed written consent was waived because only the data from the inpatient's electronic medical records were extracted.

### Data Collection

The patient's demographics, history of surgery, comorbidities, duration of UC, history of UC-related medication treatments, clinical symptoms of UC (i.e., abdominal pain, diarrhea, hematochezia, and fever), endoscopic reports, blood tests at admission, extra-intestinal manifestations, UC-related complications, UC-related medications during hospitalization, and length of stay (LOS) were manually extracted from the inpatient's electronic medical records. Fever was defined, if the body temperature, which was measured on the first day of admission according to the electronic medical records, was > 37.3°C. The Montreal classification for disease extent of UC (28) and Mayo endoscopic subscore (29) were also assessed through endoscopic reports. Several inflammatory indexes, including NLR, PLR, SII, NPR, PAR, CAR, and CLR, were calculated. NLR was calculated as the neutrophil counts (10^9^/L) divided by the lymphocyte counts (10^9^/L) (21). PLR was calculated as the platelet counts (10^9^/L) divided by the lymphocyte counts (10^9^/L) (22). SII was calculated as the neutrophil counts (10^9^/L) multiplied by the platelet counts (10^9^/L) and divided by the lymphocyte counts (10^9^/L) (23). NPR was calculated as the neutrophil counts (10^9^/L) multiplied by 1,000 and divided by the platelet counts (10^9^/L) (24). PAR was calculated as the platelet counts (10^9^/L) divided by the albumin levels (g/L) (25). CAR was calculated as the CRP levels (mg/L) divided by the albumin levels (g/L) (26). CLR was calculated as the CRP levels (mg/L) divided by the lymphocyte counts (10^9^/L) (27).

### Groups

The patients were grouped according to the activity of UC, the severity of active UC, and response to 5-ASA. First, patients were classified into active and remission UC groups according to the modified Mayo score, which has different definitions of clinical activation and remission of UC as compared to the original Mayo score (15, 29). Briefly, the modified Mayo score is calculated based on the stool frequency, rectal bleeding, endoscopic findings, and physician's global assessment. Clinical activation was defined as a total modified Mayo score of ≥3 points. Clinical remission was defined as a total modified Mayo score of ≤2 points without a sub-score of >1 point. Second, patients with active UC were classified into severe and mild-moderate UC groups according to the modified Truelove and Witts criteria (13). Severe UC was defined as a bloody stool frequency ≥6 per day along with at least one sign of systemic toxicity, including pulse rate >90 bpm, temperature > 37.8°C, hemoglobin level < 10.5 g/dl, ESR > 30 mm/h, and/or CRP > 30 mg/l. Third, patients with active UC receiving 5-ASA during their hospitalizations were classified into response and non-response to 5-ASA groups. Response to 5-ASA was defined as corticosteroid-free clinical remission, considering that corticosteroids are alternatives in our patients who did not achieve clinical remission after 5-ASA.

### Statistical Analyses

All statistical analyses were conducted using the SPSS 20.0 (SPSS Inc., Chicago, IL, United States of America), the MedCalc 20.0 (MedCalc Software bvba, Ostend, Belgium), and the GraphPad Prism 8.0.2 (GraphPad Software Inc., San Diego, CA, United States of America). Categorical data were summarized as the frequency with percentage. Differences between groups were assessed using the chi-squared test. Continuous data were summarized as mean ± SD and median with range. Differences between groups were assessed using the non-parametric Mann–Whitney U test. Spearman's correlation coefficients were used to analyze the correlation of inflammatory indexes with the activity of UC, the severity of UC, and response to 5-ASA. Correlation coefficients (r) were reported as follows: 0 < r < 1, positive correlation; −1 < r < 0, negative correlation; and r = 0, no correlation. The diagnostic accuracy of the inflammatory indexes for active UC, severe UC, and non-response to 5-ASA medications was identified by receiver operating characteristic (ROC) curves (30). Their optimal cut-off values, area under the curve (AUC) with 95% CI, sensitivity, and specificity were calculated. The optimal cutoff value was determined in the case that the summation of sensitivity and specificity values was the highest, maximizing Youden's index (31). A two-tailed *P* < 0.05 was considered statistically significant.

## Results

### Characteristics of Patients

**Figure 1:**
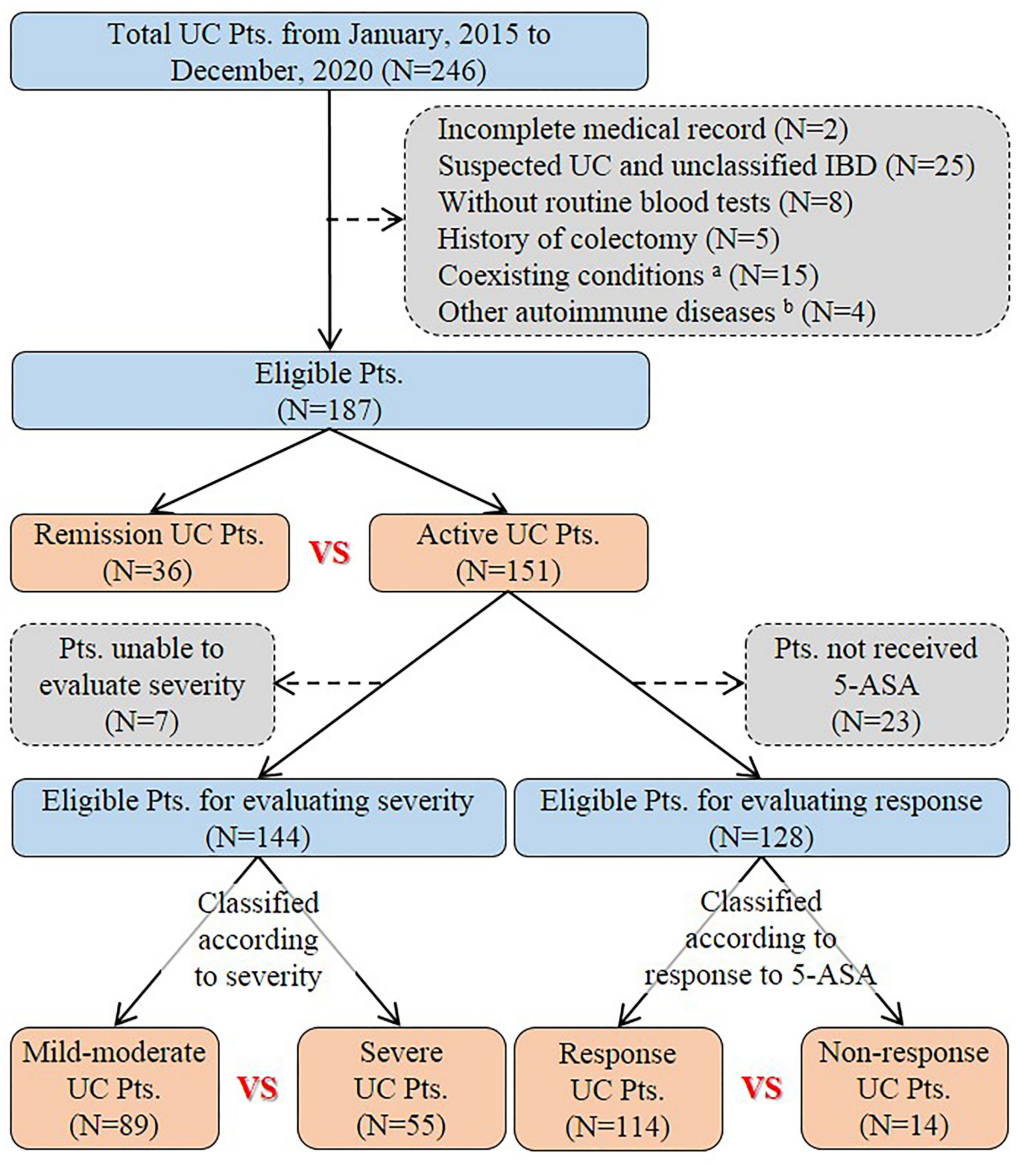
Flowchart of patient selection. ^a^Patients had coexisting conditions that potentially influence inflammatory indexes, including severe trauma (*n* = 1), pregnancy (*n* = 1), liver cirrhosis (*n* = 2), uremia (*n* = 1), and malignancy *(n* = 10). ^b^Four patients had other autoimmune diseases, including psoriasis (*n* = 1), Behcet's disease (*n* = 1), urticarial vasculitis (*n* = 1), and rheumatoid arthritis (*n* = 1). UC, Ulcerative colitis; Pts, Patients; IBD, Inflammatory bowel disease; 5-ASA, 5-aminosalicylic acid.

A total of 246 patients with UC were initially identified and reviewed for potential inclusion. Finally, 187 patients were eligible for the final analysis in the study (Figure 1). The mean age was 47.4 ± 16.3 years. The percentage of males was 53.5%. The mean disease duration was 3.6 ± 6.5 years. Nearly half of the eligible patients (51.9%) had already received 5-ASA medications for UC before admission. Diarrhea (72.2%), abdominal pain (66.8%), and hematochezia (64.7%) were the most common clinical symptoms at admission. Extensive colitis was observed in 53 (28.3%) patients through endoscopy. The Mayo endoscopic sub-score of 3 points was observed in 26 (13.9%) patients. One hundred and forty-one (75.4%) patients received 5-ASA medications for UC at our hospitals. The mean length of hospital stay was 13.2 ± 10.1 days (Table 1).

**Table 1:** Baseline characteristics of ulcerative colitis (UC) patients.

**Variables**	**No. Pts**	**Mean ±SD, Median (Range) or Frequency (Percentage)**
Age (years)	187	47.4 ± 16.3 49 (13.3–89.3)
Male (%)	187	100 (53.5%)
History of smoking (%)	187	38 (20.3%)
History of alcoholism (%)	187	17 (9.1%)
History of surgery (%)	187	52 (27.8%)
**Comorbidities**		
Hypertension/Diabetes/CHD (%)	187	14/8/10 (7.5%/4.3%/5.3%)
Duration of UC (years)	187	3.6 ± 6.5 0.5 (0–40)
**History of UC related medication treatments**		
5-ASA (%)	187	97 (51.9%)
Corticosteroids (%)	187	15 (8%)
Immunosuppressants (%)	187	1 (0.5%)
Biological agents (%)	187	1 (0.5%)
Traditional Chinese medicine (%)	187	48 (25.7%)
Probiotics (%)	187	28 (15%)
**Clinical symptoms of UC**		
Abdominal pain/Diarrhea/Hematochezia/Fever (%)	187	125/135/121/7 (66.8%/72.2%/ 64.7%/3.7%)
**Activity of UC**		
Active/Remission	187	151/36 (80.7%/19.3%)
**Severity of UC**		
Severe/Mild and moderate/Cannot be evaluated (%)	151	55/89/7 (36.4%/58.9%/4.6%)
**Montreal classification for disease extent**		
E3/E2/E1/None/Cannot be evaluated (%)	187	53/24/32/12/66 (28.3%/12.8%/17.1%/ 6.4%/35.3%)
**Mayo endoscopic subscore of UC**		
3/2/1/0/Cannot be evaluated (%)	187	26/49/7/15/90 (13.9%/26.2%/3.7%/ 8%/48.1%)
**Extra-intestinal manifestations**		
Cholelithiasis (%)	187	9 (4.8%)
Fatty liver (%)	187	10 (5.3%)
Pyoderma gangrenosum (%)	187	1 (0.5%)
Peripheral arthritis (%)	187	2 (1.1%)
**UC-related complications**		
Intestinal stenosis (%)	187	5 (2.7%)
Intestinal obstruction (%)	187	3 (1.6%)
Intraepithelial neoplasia (%)	187	15 (8%)
Massive gastrointestinal bleeding (%)	187	7 (3.7%)
**Parenteral nutrition (%)**	187	9 (4.8%)
**Central venous catheterization (%)**	187	2 (1.1%)
**UC related medication treatments during hospitalization**		
5-ASA (%)	187	141 (75.4%)
Corticosteroids (%)	187	14 (7.5%)
Biological agents (%)	187	1 (0.5%)
Traditional Chinese medicine (%)	187	69 (36.9%)
Probiotics (%)	187	107 (57.2%)
**Length of stay (days)**	187	13.2 ± 10.1 11 (1–64)

*UC, Ulcerative colitis; SD, Standard deviation; CHD, Coronary heart disease; 5-ASA, 5-aminosalicylic acid; E3, Extensive colitis; E2, Left-sided colitis; E1, Proctitis*.

### Inflammatory Indexes and Activity of UC

**Figure 2:**
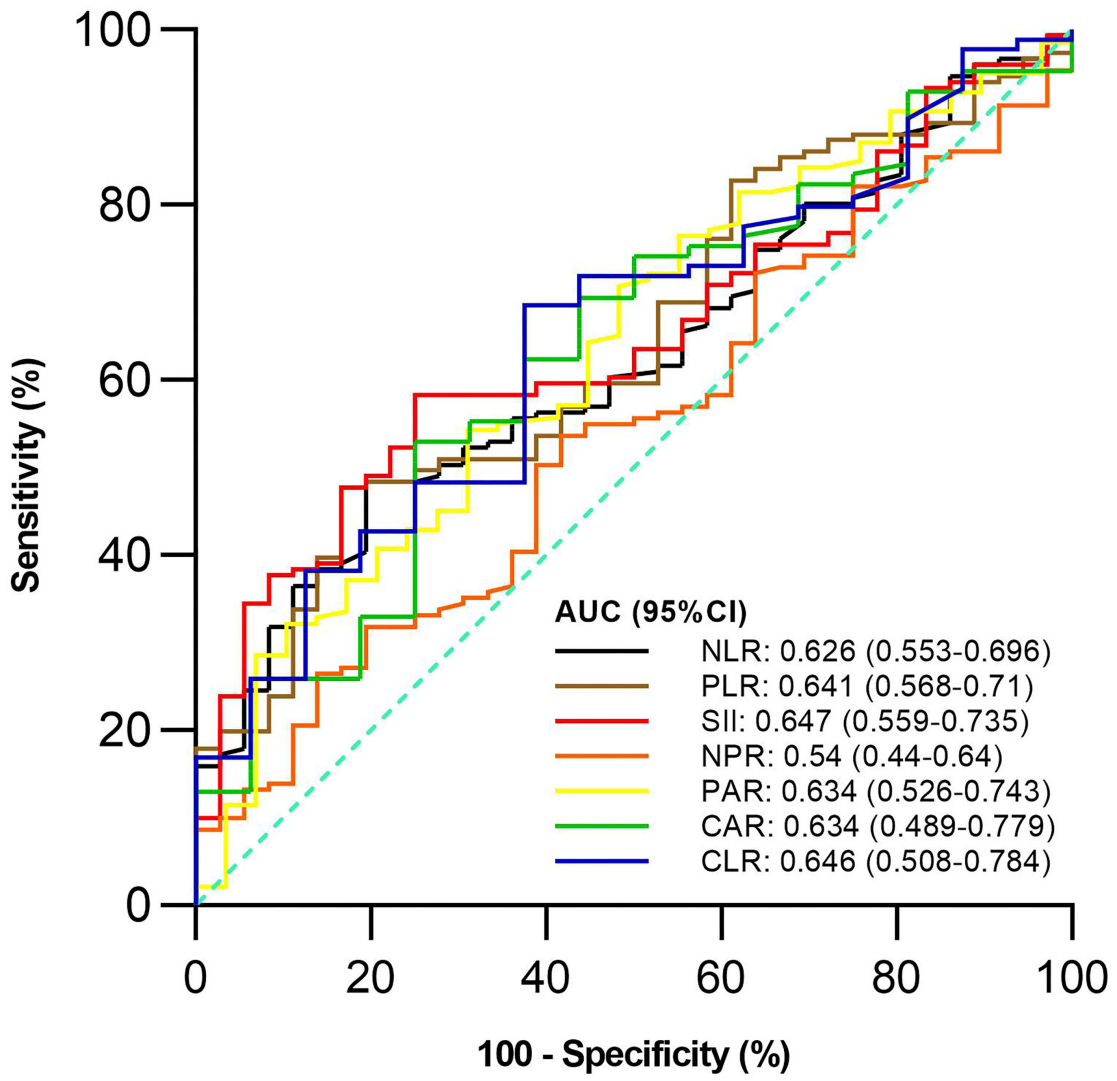
Comparison of predictive performance of inflammatory indexes for active ulcerative colitis (UC). UC, Ulcerative colitis; AUC, Area under the curve; CI, Confidence interval; NLR, Neutrophil-to-lymphocyte ratio; PLR, Platelet-to-lymphocyte ratio; SII, Systemic immune-inflammation index; NPR, Neutrophil-to-platelet ratio; PAR, Platelet-to-albumin ratio; CAR, C-reactive protein-to-albumin ratio; CLR, C-reactive protein-to-lymphocyte ratio.

Of the 187 included patients, 151 and 36 were diagnosed with active UC and remission UC, respectively. The mean NLR, PLR, SII, and PAR (*P* = 0.019, 0.009, 0.006, and 0.023), but not NPR, CAR, or CLR (*P* = 0.457, 0.091, or 0.064), were significantly higher in the active group than in the remission group (Table 2). NLR, PLR, SII, and PAR were significantly correlated with the activity of UC (*P* = 0.02, 0.01, 0.01, and 0.02) (Table 3). SII had the largest AUC (AUC = 0.647), followed by CLR, PLR, PAR, CAR, NLR, and NPR (AUC = 0.646, 0.641, 0.634, 0.634, 0.626, and 0.54) (Figure 2). The optimal cut-off value of SII for active UC was 595.47 × 10^9^/L with a sensitivity and specificity of 58.28 and 75%, respectively (Supplementary Table 1). The AUC of SII was significantly different from that of NPR (*P* = 0.0428), but not NLR, PLR, PAR, CAR, or CLR (*P* = 0.3506, 0.8508, 0.8233, 0.5929, or 0.6023).

**Table 2:** Comparison of inflammatory indexes between active and remission UC groups.

**Indexes**	**Active group**		**Remission group**		***P*-value**
	**No. Pts**	**Mean ±SD, Median (Range) or Frequency (Percentage)**	**No. Pts**	**Mean ±SD, Median (Range) or Frequency (Percentage)**	
NLR	151	3.53 ± 3.16 2.63 (0.63–20.33)	36	2.23 ± 1.06 2 (0.88–5.3)	**0.019**
PLR	151	181.17 ± 103.53 153.08 (46.25–632.22)	36	133.75 ± 47.45 136.84 (63.55–240)	**0.009**
SII	151	1117.49 ± 1428.01 676 (105–10558.11)	36	552.34 ± 397.31 517.76 (150–2400)	**0.006**
NPR	151	19.33 ± 10.66 17.06 (5.25–83.82)	36	17.13 ± 6.21 15.87 (7.58–31.36)	0.457
PAR	140	8.02 ± 4.39 6.54 (1.57–29.03)	29	6.37 ± 3.33 5.4 (2.86–19.65)	**0.023**
CAR	85	0.98 ± 2.01 0.21 (0.003–14.79)	16	0.33 ± 0.59 0.04 (0.006–2.14)	0.091
CLR	89	21.46 ± 41.9 3.89 (0.06–322)	16	5.9 ± 10.72 0.78 (0.08–38.95)	0.064

*UC, Ulcerative colitis; Pts, Patients; SD, Standard deviation; NLR, Neutrophil-to-lymphocyte ratio; PLR, Platelet-to-lymphocyte ratio; SII, Systemic immune-inflammation index; NPR, Neutrophil-to-platelet ratio; PAR, Platelet-to-albumin ratio; CAR, C-reactive protein-to-albumin ratio; CLR, C-reactive protein-to-lymphocyte ratio*.

**Table 3:** Correlation analyses of inflammatory indexes with the activity of UC, the severity of UC, and non-response to 5-aminosalicylic acid (5-ASA).

**Indexes**	**Activity of UC**		**Severity of UC**		**Non-response to 5-ASA**	
	***r***	***P*-value**	***r***	***P*-value**	***r***	***P*-value**
NLR	0.172	**0.02**	0.245	**<0.001**	0.137	0.123
PLR	0.193	**0.01**	0.324	**<0.001**	0.097	0.277
SII	0.201	**0.01**	0.284	**<0.001**	0.148	0.096
NPR	0.055	0.459	0.042	0.618	0.12	0.176
PAR	0.175	**0.02**	0.219	**0.01**	0.159	0.083
CAR	0.169	0.091	0.369	**<0.001**	0.353	**0.002**
CLR	0.182	0.063	0.401	**<0.001**	0.321	**0.004**

*UC, Ulcerative colitis; 5-ASA, 5-aminosalicylic acid; NLR, Neutrophil-to-lymphocyte ratio; PLR, Platelet-to-lymphocyte ratio; SII, Systemic immune-inflammation index; NPR, Neutrophil-to-platelet ratio; PAR, Platelet-to-albumin ratio; CAR, C-reactive protein-to-albumin ratio; CLR, C-reactive protein-to-lymphocyte ratio*.

### Inflammatory Indexes and Severity of UC

**Figure 3:**
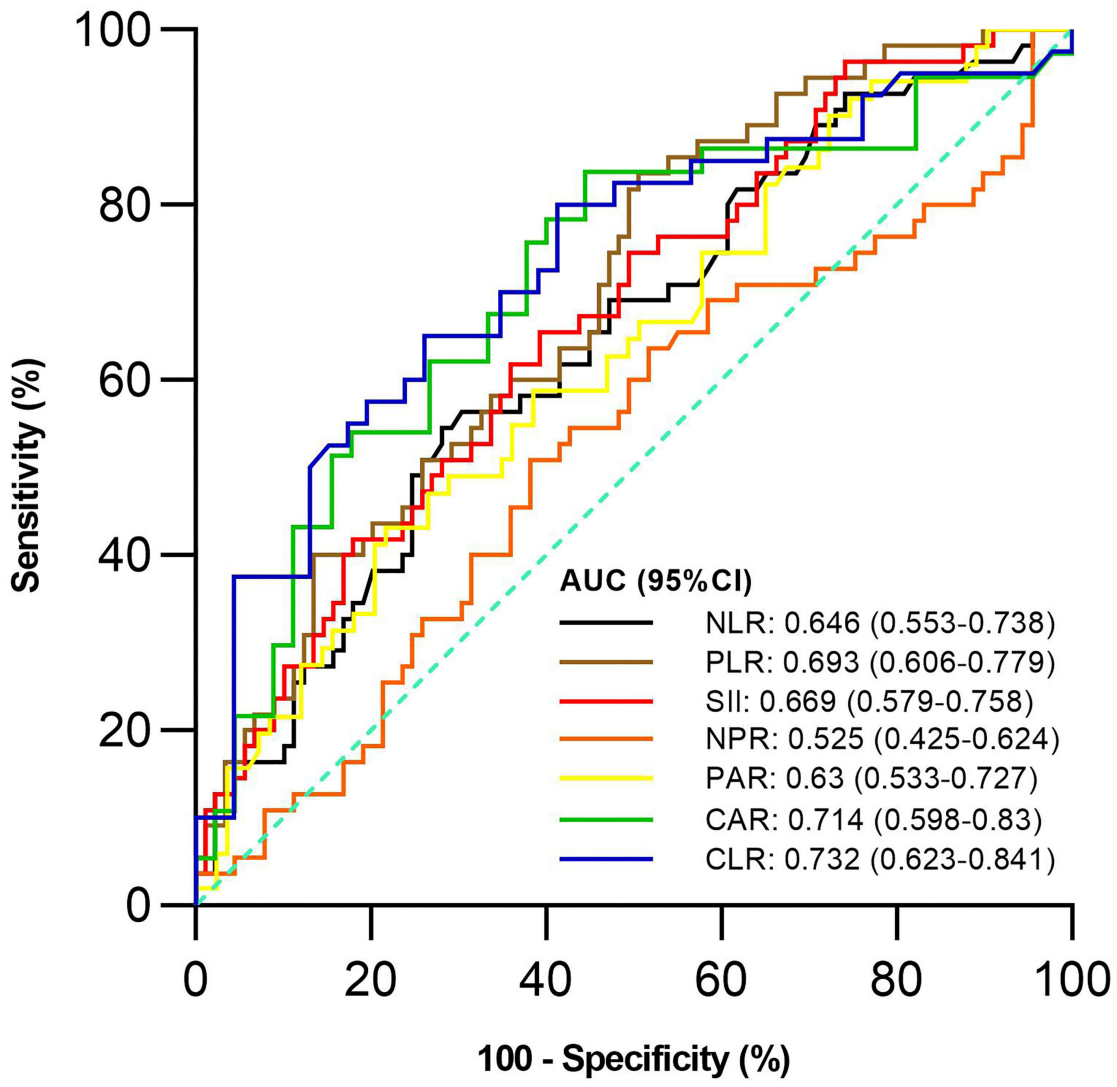
Comparison of the predictive performance of inflammatory indexes for severe UC. UC, Ulcerative colitis; AUC, Area under the curve; CI, Confidence interval; NLR, Neutrophil-to-lymphocyte ratio; PLR, Platelet-to-lymphocyte ratio; SII, Systemic immune-inflammation index; NPR, Neutrophil-to-platelet ratio; PAR, Platelet-to-albumin ratio; CAR, C-reactive protein-to-albumin ratio; CLR, C-reactive protein-to-lymphocyte ratio.

Of the 144 active UC patients, 55 and 89 were diagnosed with severe UC and mild-moderate UC, respectively. The mean NLR, PLR, SII, PAR, CAR, and CLR (*P* = 0.003, <0.001, 0.001, 0.012, 0.001, and <0.001), but not NPR (*P* = 0.616), were significantly higher in the severe group than in the mild-moderate group (Table 4). NLR, PLR, SII, PAR, CAR, and CLR were significantly correlated with the severity of UC (*P* =< 0.001, <0.001, <0.001, 0.01, <0.001, and <0.001) (Table 3). CLR had the largest AUC (AUC = 0.732), followed by CAR, PLR, SII, NLR, PAR, and NPR (AUC = 0.714, 0.693, 0.669, 0.646, 0.63, and 0.525) (Figure 3). The optimal cut-off value of CLR for severe UC was 7 mg/10^9^ with a sensitivity and specificity of 65% and 73.91%, respectively (Supplementary Table 2). The AUC of CLR was significantly different from that of SII, NPR, and PAR (*P* = 0.0473, 0.043, and 0.0138), but not NLR, PLR, or CAR (*P* = 0.0562, 0.0723, or 0.1886).

**Table 4:** Comparison of inflammatory indexes between severe and mild-moderate UC groups.

**Indexes**	**Severe group**		**Mild-moderate group**		***P*-value**
	**No. Pts**	**Mean ±SD, Median (Range) or Frequency (Percentage)**	**No. Pts**	**Mean ±SD, Median (Range) or Frequency (Percentage)**	
NLR	55	4.48 ± 4.02 3.27 (1–20.33)	89	2.92 ± 2.35 2.14 (0.63–15.5)	**0.003**
PLR	55	221.57 ± 124.25 183 (81.76–632.22)	89	156.73 ± 80.9 131.69 (46.25–488.89)	**<0.001**
SII	55	1587.16 ± 1973.78 910 (212.59–10558.11)	89	844.4 ± 909.58 552 (105–6045)	**0.001**
NPR	55	19.91 ± 12.63 18.1 (6.62–83.82)	89	18.78 ± 9.46 16.11 (5.25-50.99)	0.616
PAR	51	9.27 ± 4.77 7.42 (3.7–29.03)	83	7.44 ± 4.1 6.17 (1.57–27.74)	**0.012**
CAR	37	1.64 ± 2.69 0.76 (0.003–14.79)	45	0.51 ± 1.04 0.06 (0.01–5.13)	**0.001**
CLR	40	36.72 ± 55.78 20.08 (0.06–322)	46	9.51 ± 18.9 1.22 (0.09–87.06)	**<0.001**

*UC, Ulcerative colitis; Pts, Patients; SD, Standard deviation; NLR, Neutrophil-to-lymphocyte ratio; PLR, Platelet-to-lymphocyte ratio; SII, Systemic immune-in?ammation index; NPR, Neutrophil-to-platelet ratio; PAR, Platelet-to-albumin ratio; CAR, C-reactive protein-to-albumin ratio; CLR, C-reactive protein-to-lymphocyte ratio*.

### Inflammatory Indexes and Non-Response to 5-ASA

**Figure 4:**
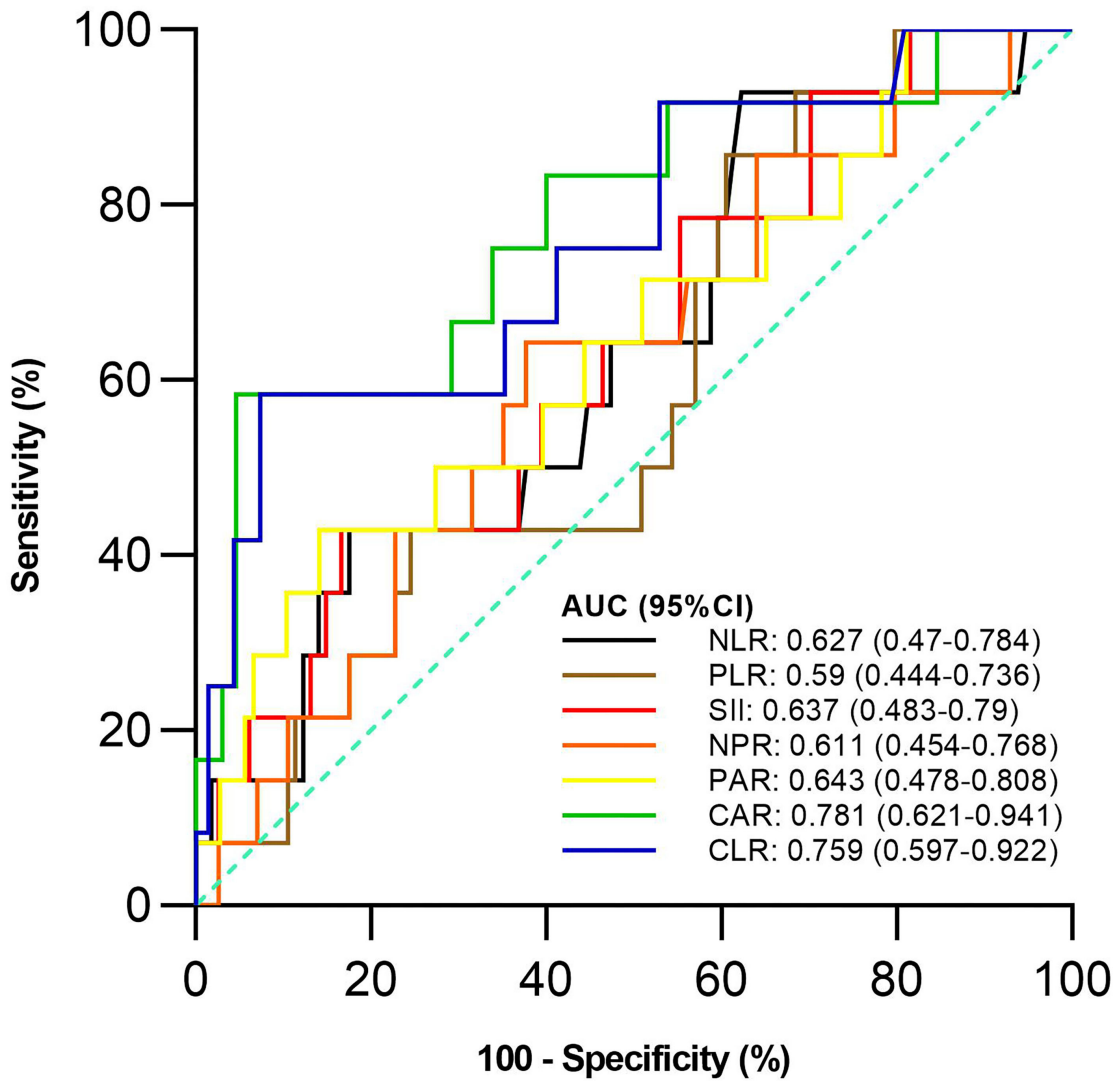
Comparison of the predictive performance of inflammatory indexes for non-response to 5-aminosalicylic acid (5-ASA). 5-ASA, 5-aminosalicylic acid; AUC, Area under the curve; CI, Confidence interval; NLR, Neutrophil-to-lymphocyte ratio; PLR, Platelet-to-lymphocyte ratio; SII, Systemic immune-inflammation index; NPR, Neutrophil-to-platelet ratio; PAR, Platelet-to-albumin ratio; CAR, C-reactive protein-to-albumin ratio; CLR, C-reactive protein-to-lymphocyte ratio.

Of the 128 active UC patients who received 5-ASA, 14 were not responsive to 5-ASA. The mean CAR and CLR (*P* = 0.002 and 0.004), but not NLR, PLR, SII, NPR, and PAR (*P* = 0.122, 0.275, 0.096, 0.175, and 0.083), were significantly higher in the non-response group than in the response group (Table 5). CAR and CLR were significantly correlated with non-response to 5-ASA (*P* = 0.002 and 0.004) (Table 3). CAR had the largest AUC (AUC = 0.781), followed by CLR, PAR, SII, NLR, NPR, and PLR (AUC = 0.759, 0.643, 0.637, 0.627, 0.611, and 0.59) (Figure 4). The optimal cut-off value of CAR for non-response to 5-ASA was 2.41mg/g with a sensitivity and specificity of 58.33% and 95.38%, respectively (Supplementary Table 3). The AUC of CAR was significantly different from that of PLR (*P* = 0.0014), but not NLR, SII, NPR, PAR, or CLR (*P* = 0.055, 0.0673, 0.1451, 0.1529, or 0.3271).

**Table 5:** Comparison of inflammatory indexes between non-response and response to 5-ASA groups.

**Indexes**	**Non-response group**		**Response group**		***P*-value**
	**No. Pts**	**Mean ±SD, Median (Range) or Frequency (Percentage)**	**No. Pts**	**Mean ±SD, Median (Range) or Frequency (Percentage)**	
NLR	14	4.99 ± 4.85 2.94 (1.13–18.56)	114	3.24 ± 2.57 2.56 (0.63–15.5)	0.122
PLR	14	213.15 ± 134.4 154.04 (109.41–632.22)	114	180.99 ± 102.05 163.44 (54.62–618.75)	0.275
SII	14	1953.09 ± 2716.53 841.01 (304–10558.11)	114	990.99 ± 1025.29 659.97 (105–6045)	0.096
NPR	14	20.95 ± 8.99 19.72 (8.03–41.97)	114	18.14 ± 8.86 16.28 (5.25–50.99)	0.175
PAR	14	10.89 ± 6.65 8.47 (4.87–29.03)	106	7.97 ± 4.11 6.57 (2.73–27.74)	0.083
CAR	12	3.21 ± 4.16 2.71 (0.01–14.79)	65	0.61 ± 1.07 0.14 (0.003–5.13)	**0.002**
CLR	12	65.78 ± 88.51 56.63 (0.29–322)	68	14.21 ± 23.26 3.18 (0.06–115)	**0.004**

*5-ASA, 5-aminosalicylic acid; Pts, Patients; SD, Standard deviation; NLR, Neutrophil-to-lymphocyte ratio; PLR, Platelet-to-lymphocyte ratio; SII, Systemic immune-inflammation index; NPR, Neutrophil-to-platelet ratio; PAR, Platelet-to-albumin ratio; CAR, C-reactive protein-to-albumin ratio; CLR, C-reactive protein-to-lymphocyte ratio*.

## Discussion

The present study has compared the value of seven inflammatory indexes, including NLR, PLR, SII, NPR, PAR, CAR, and CLR, at baseline for assessing the activity of UC, the severity of UC, and response to 5-ASA, and found that SII had the greatest predictive value for active UC, CLR for severe UC, and CAR for non-response to 5-ASA.

Systemic immune-inflammation index (SII) was originally developed as an independent predictor of recurrence and survival for patients with hepatocellular carcinoma after surgery (23). Recently, it has also been demonstrated that SII level was higher in patients with UC than in healthy control groups and that SII level was positively associated with disease activity in patients (32) with UC. Similarly, our study found that the SII level was significantly correlated with the activity of UC. Moreover, SII had a better diagnostic capability for active UC than other inflammatory indexes. This finding may be attributed to the fact that immunity and inflammation are crucial for the occurrence of UC (1) and SII is calculated as neutrophil counts multiplied by platelet counts and divided by lymphocyte counts (23).

First, in a healthy human body, although 1-2 × 10^11^ neutrophils are generated every day in the bone marrow (33), chemokine receptors, including CXCR4 and CXCR2, may maintain a delicate balance of neutrophil counts by mediating the retention of neutrophils in the bone marrow and their mobilization to peripheral blood (34, 35). In patients with active inflammatory bowel disease (IBD), there are significantly increased interleukin-17A (IL-17A) levels in inflamed mucosa that promote the transcription of granulocyte colony-stimulating factor in bone marrow (36), thereby inhibiting and activating the expression of CXCR4 binding ligands and CXCR2 binding ligands, respectively. As a result, increased neutrophils are released from the bone marrow into the peripheral blood (37). Therefore, patients with active IBD often have peripheral neutrophilia (38).

Second, lymphocytes, such as Th1 cells, Th17 cells, and B cells, can produce pro-inflammatory cytokines and activate intestinal proteases leading to mucosal damage (39), and are accumulated in the inflamed lamina propria of IBD (40, 41). In patients with active IBD, lymphocytes are accumulated from the peripheral blood into the inflamed intestine, which eventually results in peripheral lymphopenia (39).

Third, platelets are produced from long cytoplasmic processes fragmentation of megakaryocyte in the extravascular marrow space (42). Patients with IBD have elevated levels of thrombopoietin and IL-6, which are involved in megakaryocytic maturation (43). Moreover, in patients with IBD, the platelets in the peripheral blood are active and can spontaneously aggregate with increased susceptibility to proaggregating agents (44). Thus, patients with active UC often have peripheral thrombocytosis (45). Additionally, platelet counts are more strongly associated with the activity of UC than the severity of UC. Platelet counts in peripheral blood are markedly increased in patients with active UC compared with inactive UC (45), but not correlated with the Ulcerative Colitis Colonoscopic Index of Severity, which has an excellent overall assessment of endoscopic severity (46).

C-reactive protein-to-lymphocyte ratio (CLR) was originally developed as a novel index to predict the major morbidity after esophagogastric cancer resection (27). To our knowledge, only Con et al. explored the role of CLR for assessing the dynamic response to infliximab salvage treatment and predicting the risk of subsequent colectomy in patients with UC (47). Similarly, our study found that CLR had a better capability to predict the severity of UC than other inflammatory indexes. This finding may be attributed to the fact that CLR is calculated as CRP levels divided by lymphocyte counts (27). CRP, the most important acute-phase protein, is produced almost exclusively by hepatocytes in response to stimulation by IL-6, IL-1β, and tumor necrosis factor α. In the presence of an acute-phase inflammation or infection, CRP levels are increased dramatically. Contrarily, CRP levels are quickly decreased when inflammation is effectively treated (48). CRP alone can be used to predict the severity of active UC (49). Additionally, CRP levels are more strongly associated with the severity of UC than the activity of UC (50).

C-reactive protein-to-albumin ratio (CAR) was originally developed to predict the outcome of patients from acute medical ward (26). Gibson et al. (51) detected the predictive value of CAR to steroid response in patients with acute severe UC aiming to select patients who need early rescue treatment (51). Similarly, our study found that CAR had a better capability to predict the response to 5-ASA than other inflammatory indexes. This finding may be attributed to the fact that CAR is calculated as CRP levels divided by albumin levels (26). First, CRP levels are significantly correlated with the proportion of corticosteroid use in patients (52) with Crohn's disease. Corticosteroids, as rescue medications, are usually used in patients who are not well responsive to 5-ASA (9, 10). Second, albumin levels can reflect the nutritional status of patients (53) with UC and are correlated significantly with the clinical severity of UC (54). Moreover, an *in vitro* study suggested a strong interaction between 5-ASA and human serum albumin (55). In a mouse model of UC, 5-ASA conjugated with human serum albumin was found to show a significant therapeutic effect (56). Thus, albumin levels may influence the therapeutic effect of 5-ASA for UC.

The present study had several limitations. First, it was conducted at a single center with a relatively small sample size of total patients and a very small sample size of patients in the non-response group, probably compromising the statistical analyses presented. Second, the external validity of our findings was lacking. Third, endoscopy was not performed by the same expert at our hospital, so endoscopic assessment might be a bit inconsistent. Fourth, this study was retrospective and cross-sectional, where CRP and albumin levels were not routinely tested in our patients. Moreover, there were some damages in the inpatient information system, leading to the lack of details of two patient's medical records. Fifth, only a few patients were treated with corticosteroids or biologics in our study. Therefore, we cannot evaluate the correlation between inflammatory indexes and response to corticosteroids or biologics.

In conclusion, SII, CLR, and CAR have higher diagnostic performance than other inflammatory indexes for active UC, severe UC, and response to 5-ASA, respectively. Dynamic changes of these inflammatory indexes along with activity and severity of UC should be further explored. Moreover, future studies should also evaluate the association between these inflammatory indexes and mucosal severity of UC.

## Data Availability Statement

The original contributions presented in the study are included in the article/Supplementary Material, further inquiries can be directed to the corresponding authors.

## Ethics Statement

The studies involving human participants were reviewed and approved by Ethical Committee of General Hospital of Northern Theater Command. Written informed consent from the participants' legal guardian/next of kin was not required to participate in this study in accordance with the national legislation and the institutional requirements.

## Author Contributions

XQ: conceptualization. HL and XQ: methodology. HL, ZB, and XQ: formal analysis and writing–original draft. HL, ZB, GC, and XQ: data curation. HL, ZB, QW, GC, YZ, XG, and XQ: writing–review and editing. XG and XQ: supervision. All authors have made an intellectual contribution to the manuscript and approved the submission.

## Funding

YZ received funding supported by the Natural Science Foundation of Liaoning Province (No. 2019-ZD-1058).

## Conflict of Interest

The authors declare that the research was conducted in the absence of any commercial or financial relationships that could be construed as a potential conflict of interest.

## Publisher's Note

All claims expressed in this article are solely those of the authors and do not necessarily represent those of their affiliated organizations, or those of the publisher, the editors and the reviewers. Any product that may be evaluated in this article, or claim that may be made by its manufacturer, is not guaranteed or endorsed by the publisher.
