# Supplementary material for: Inflammatory Indexes for Assessing the Severity and Disease Progression of Ulcerative Colitis: A Single-Center Retrospective Study
Source: Front Public Health. 2022 Mar 10;10:851295. doi: 10.3389/fpubh.2022.851295 (PMC8963422; doi:10.3389/fpubh.2022.851295)
Supplement: Supplementary file 1 [file Table_1.DOCX]

| **Supplementary table 1. Receiver operating characteristic analyses of inflammatory indexes in distinguishing active UC** | | | | | |
| --- | --- | --- | --- | --- | --- |
| **Indexes** | **Cut-off** | **AUC (95%CI)** | **Sensitivity** | **Specificity** | **P-value** |
| NLR | 2.69 | 0.626 (0.553-0.696) | 48.34% | 80.56% | 0.0067 |
| PLR | 167.86 | 0.641 (0.568-0.71) | 47.68% | 83.33% | 0.003 |
| SII | 595.47 | 0.647 (0.559-0.735) | 58.28% | 75% | 0.001 |
| NPR | 23.36 | 0.54 (0.44-0.64) | 26.49% | 86.11% | 0.4322 |
| PAR | 6.23 | 0.634 (0.526-0.743) | 54.29% | 68.97% | 0.0154 |
| CAR | 0.1779 | 0.634 (0.489-0.779) | 52.94% | 75% | 0.0706 |
| CLR | 1 | 0.646 (0.508-0.784) | 68.54% | 62.5% | 0.0382 |
| **Abbreviations:** UC, Ulcerative colitis; AUC, Area under the curve; CI, Confidence interval; NLR, Neutrophil-to-lymphocyte ratio; PLR, Platelet-to-lymphocyte ratio; SII, Systemic immune-inﬂammation index; NPR, Neutrophil-to-platelet ratio; PAR, Platelet-to-albumin ratio; CAR, C-reactive protein-to-albumin ratio; CLR, C-reactive protein-to-lymphocyte ratio. | | | | | |
