# Supplementary material for: Inflammatory Indexes for Assessing the Severity and Disease Progression of Ulcerative Colitis: A Single-Center Retrospective Study
Source: Front Public Health. 2022 Mar 10;10:851295. doi: 10.3389/fpubh.2022.851295 (PMC8963422; doi:10.3389/fpubh.2022.851295)
Supplement: Supplementary file 2 [file Table_2.DOCX]

| **Supplementary table 2. Receiver operating characteristic analyses of inflammatory indexes in distinguishing severe UC** | | | | | |
| --- | --- | --- | --- | --- | --- |
| **Indexes** | **Cut-off** | **AUC (95%CI)** | **Sensitivity** | **Specificity** | **P-value** |
| NLR | 3.08 | 0.646 (0.553-0.738) | 54.55% | 71.91% | 0.002 |
| PLR | 130 | 0.693 (0.606-0.779) | 83.64% | 49.44% | <0.0001 |
| SII | 676.5 | 0.669 (0.579-0.758) | 65.45% | 60.67% | 0.0002 |
| NPR | 18.07 | 0.525 (0.425-0.624) | 50.91% | 61.8% | 0.6237 |
| PAR | 9.18 | 0.63 (0.533-0.727) | 43.14% | 78.31% | 0.0083 |
| CAR | 0.0961 | 0.714 (0.598-0.83) | 83.78% | 55.56% | 0.0003 |
| CLR | 7 | 0.732 (0.623-0.841) | 65% | 73.91% | <0.0001 |
| **Abbreviations:** UC, Ulcerative colitis; AUC, Area under the curve; CI, Confidence interval; NLR, Neutrophil-to-lymphocyte ratio; PLR, Platelet-to-lymphocyte ratio; SII, Systemic immune-inﬂammation index; NPR, Neutrophil-to-platelet ratio; PAR, Platelet-to-albumin ratio; CAR, C-reactive protein-to-albumin ratio; CLR, C-reactive protein-to-lymphocyte ratio. | | | | | |
