# Supplementary material for: Inflammatory Indexes for Assessing the Severity and Disease Progression of Ulcerative Colitis: A Single-Center Retrospective Study
Source: Front Public Health. 2022 Mar 10;10:851295. doi: 10.3389/fpubh.2022.851295 (PMC8963422; doi:10.3389/fpubh.2022.851295)
Supplement: Supplementary file 3 [file Table_3.DOCX]

| **Supplementary table 3. Receiver operating characteristic analyses of inflammatory indexes in distinguishing non-response to 5-ASA** | | | | | |
| --- | --- | --- | --- | --- | --- |
| **Indexes** | **Cut-off** | **AUC (95%CI)** | **Sensitivity** | **Specificity** | **P-value** |
| NLR | 1.86 | 0.627 (0.47-0.784) | 92.86% | 37.72% | 0.1132 |
| PLR | 133.33 | 0.59 (0.444-0.736) | 85.71% | 39.47% | 0.2292 |
| SII | 1443.08 | 0.637 (0.483-0.79) | 42.86% | 83.33% | 0.0817 |
| NPR | 18.33 | 0.611 (0.454-0.768) | 64.29% | 62.28% | 0.1653 |
| PAR | 12.16 | 0.643 (0.478-0.808) | 42.86% | 85.85% | 0.09 |
| CAR | 2.4131 | 0.781 (0.621-0.941) | 58.33% | 95.38% | 0.0006 |
| CLR | 54.25 | 0.759 (0.597-0.922) | 58.33% | 92.65% | 0.0017 |
| **Abbreviations:** 5-ASA, 5-aminosalicylic acid; AUC, Area under the curve; CI, Confidence interval; NLR, Neutrophil-to-lymphocyte ratio; PLR, Platelet-to-lymphocyte ratio; SII, Systemic immune-inﬂammation index; NPR, Neutrophil-to-platelet ratio; PAR, Platelet-to-albumin ratio; CAR, C-reactive protein-to-albumin ratio; CLR, C-reactive protein-to-lymphocyte ratio. | | | | | |
