# Supplementary material for: Inflammatory Indexes for Assessing the Severity and Disease Progression of Ulcerative Colitis: A Single-Center Retrospective Study
Source: Front Public Health. 2022 Mar 10;10:851295. doi: 10.3389/fpubh.2022.851295 (PMC8963422; doi:10.3389/fpubh.2022.851295)
Supplement: Supplementary file 4 [file Data_Sheet_1.docx]

| **Table 1. Receiver operating characteristic analyses of inflammatory indexes in distinguishing active UC for male patients** | | | | | |
| --- | --- | --- | --- | --- | --- |
| **Indexes** | **Cut-off** | **AUC (95%CI)** | **Sensitivity** | **Specificity** | **P-value** |
| NLR | 2.69 | 0.655 (0.554-0.747) | 49.4% | 88.24% | 0.0171 |
| PLR | 167.86 | 0.661 (0.559-0.752) | 43.37% | 88.24% | 0.024 |
| SII | 595.47 | 0.689 (0.589-0.778) | 59.04% | 88.24% | 0.0011 |
| NPR | 11.71 | 0.526 (0.424-0.627) | 87.95% | 29.41% | 0.7516 |
| PAR | 6.23 | 0.727 (0.619-0.818) | 55.41% | 90.91% | 0.0011 |
| CAR | 0.0293 | 0.727 (0.592-0.838) | 76.47% | 80.00% | 0.0384 |
| CLR | 0.76 | 0.77 (0.642-0.870) | 72.22% | 80.00% | 0.0083 |
| **Abbreviations:** UC, Ulcerative colitis; AUC, Area under the curve; CI, Confidence interval; NLR, Neutrophil-to-lymphocyte ratio; PLR, Platelet-to-lymphocyte ratio; SII, Systemic immune-inﬂammation index; NPR, Neutrophil-to-platelet ratio; PAR, Platelet-to-albumin ratio; CAR, C-reactive protein-to-albumin ratio; CLR, C-reactive protein-to-lymphocyte ratio. | | | | | |

| **Table 2. Receiver operating characteristic analyses of inflammatory indexes in distinguishing active UC for female patients** | | | | | |
| --- | --- | --- | --- | --- | --- |
| **Indexes** | **Cut-off** | **AUC (95%CI)** | **Sensitivity** | **Specificity** | **P-value** |
| NLR | 2.69 | 0.597 (0.486-0.701) | 47.06% | 73.68% | 0.155 |
| PLR | 163 | 0.631 (0.521-0.732) | 54.41% | 78.95% | 0.0476 |
| SII | 940.08 | 0.596 (0.485-0.700) | 30.88% | 94.74% | 0.1649 |
| NPR | 23.36 | 0.543 (0.433-0.650) | 30.88% | 94.74% | 0.5172 |
| PAR | 5.4 | 0.581 (0.430-0.732) | 69.70% | 50.00% | 0.2936 |
| CAR | 0.9646 | 0.561 (0.364-0.759) | 26.47% | 90.91% | 0.5418 |
| CLR | 1 | 0.562 (0.370-0.755) | 68.57% | 54.55% | 0.5249 |
| **Abbreviations:** UC, Ulcerative colitis; AUC, Area under the curve; CI, Confidence interval; NLR, Neutrophil-to-lymphocyte ratio; PLR, Platelet-to-lymphocyte ratio; SII, Systemic immune-inﬂammation index; NPR, Neutrophil-to-platelet ratio; PAR, Platelet-to-albumin ratio; CAR, C-reactive protein-to-albumin ratio; CLR, C-reactive protein-to-lymphocyte ratio. | | | | | |

| **Table 3. Receiver operating characteristic analyses of inflammatory indexes in distinguishing severe UC for male patients** | | | | | |
| --- | --- | --- | --- | --- | --- |
| **Indexes** | **Cut-off** | **AUC (95%CI)** | **Sensitivity** | **Specificity** | **P-value** |
| NLR | 3.08 | 0.699 (0.586-0.797) | 64.52% | 72.92% | 0.0007 |
| PLR | 130 | 0.685 (0.571-0.785) | 87.10% | 47.92% | 0.0018 |
| SII | 676.5 | 0.704 (0.590-0.801) | 74.19% | 60.42% | 0.0005 |
| NPR | 18.07 | 0.611 (0.495-0.719) | 61.29% | 64.58% | 0.0938 |
| PAR | 5.18 | 0.649 (0.526-0.758) | 96.30% | 38.64% | 0.0222 |
| CAR | 0.5748 | 0.744 (0.599-0.858) | 60.87% | 84.62% | 0.0008 |
| CLR | 10.92 | 0.746 (0.606-0.856) | 65.38% | 80.77% | 0.0006 |
| **Abbreviations:** UC, Ulcerative colitis; AUC, Area under the curve; CI, Confidence interval; NLR, Neutrophil-to-lymphocyte ratio; PLR, Platelet-to-lymphocyte ratio; SII, Systemic immune-inﬂammation index; NPR, Neutrophil-to-platelet ratio; PAR, Platelet-to-albumin ratio; CAR, C-reactive protein-to-albumin ratio; CLR, C-reactive protein-to-lymphocyte ratio. | | | | | |

| **Table 4. Receiver operating characteristic analyses of inflammatory indexes in distinguishing severe UC for female patients** | | | | | |
| --- | --- | --- | --- | --- | --- |
| **Indexes** | **Cut-off** | **AUC (95%CI)** | **Sensitivity** | **Specificity** | **P-value** |
| NLR | 5 | 0.573 (0.444-0.695) | 29.17% | 92.68% | 0.3475 |
| PLR | 213.08 | 0.702 (0.576-0.809) | 45.83% | 90.24% | 0.0034 |
| SII | 1123.73 | 0.614 (0.485-0.732) | 37.50% | 87.80% | 0.1261 |
| NPR | 9.29 | 0.559 (0.430-0.682) | 33.33% | 87.80% | 0.442 |
| PAR | 8.46 | 0.612 (0.481-0.732) | 54.17% | 82.05% | 0.157 |
| CAR | 0.1337 | 0.684 (0.500-0.834) | 71.43% | 73.68% | 0.0696 |
| CLR | 2.31 | 0.711 (0.530-0.853) | 71.43% | 65.00% | 0.0285 |
| **Abbreviations:** UC, Ulcerative colitis; AUC, Area under the curve; CI, Confidence interval; NLR, Neutrophil-to-lymphocyte ratio; PLR, Platelet-to-lymphocyte ratio; SII, Systemic immune-inﬂammation index; NPR, Neutrophil-to-platelet ratio; PAR, Platelet-to-albumin ratio; CAR, C-reactive protein-to-albumin ratio; CLR, C-reactive protein-to-lymphocyte ratio. | | | | | |

| **Table 5. Receiver operating characteristic analyses of inflammatory indexes in distinguishing non-response to 5-ASA for male patients** | | | | | |
| --- | --- | --- | --- | --- | --- |
| **Indexes** | **Cut-off** | **AUC (95%CI)** | **Sensitivity** | **Specificity** | **P-value** |
| NLR | 1.83 | 0.659 (0.537-0.767) | 100.00% | 36.07% | 0.0614 |
| PLR | 119.57 | 0.592 (0.469-0.707) | 100.00% | 29.51% | 0.2744 |
| SII | 1443.08 | 0.639 (0.517-0.750) | 50.00% | 81.97% | 0.1466 |
| NPR | 18.33 | 0.663 (0.541-0.771) | 70.00% | 63.93% | 0.0792 |
| PAR | 12.13 | 0.633 (0.504-0.749) | 40.00% | 87.27% | 0.1873 |
| CAR | 2.1145 | 0.776 (0.630-0.884) | 60.00% | 97.30% | 0.0048 |
| CLR | 38.94 | 0.741 (0.596-0.856) | 60.00% | 92.31% | 0.0158 |
| **Abbreviations:** 5-ASA, 5-aminosalicylic acid; AUC, Area under the curve; CI, Confidence interval; NLR, Neutrophil-to-lymphocyte ratio; PLR, Platelet-to-lymphocyte ratio; SII, Systemic immune-inﬂammation index; NPR, Neutrophil-to-platelet ratio; PAR, Platelet-to-albumin ratio; CAR, C-reactive protein-to-albumin ratio; CLR, C-reactive protein-to-lymphocyte ratio. | | | | | |

| **Table 6. Receiver operating characteristic analyses of inflammatory indexes in distinguishing non-response to 5-ASA for female patients** | | | | | |
| --- | --- | --- | --- | --- | --- |
| **Indexes** | **Cut-off** | **AUC (95%CI)** | **Sensitivity** | **Specificity** | **P-value** |
| NLR | 11.13 | 0.533 (0.396-0.666) | 25.00% | 100.00% | 0.8591 |
| PLR | 618.75 | 0.575 (0.437-0.705) | 25.00% | 100.00% | 0.6788 |
| SII | 375.39 | 0.618 (0.480-0.743) | 100.00% | 30.19% | 0.4517 |
| NPR | 29.09 | 0.524 (0.387-0.658) | 25.00% | 90.57% | 0.888 |
| PAR | 14.09 | 0.681 (0.542-0.801) | 50.00% | 92.16% | 0.3273 |
| CAR | 0.1912 | 0.839 (0.660-0.947) | 100.00% | 67.86% | 0.042 |
| CLR | 2.31 | 0.776 (0.591-0.905) | 100.00% | 55.17% | 0.2284 |
| **Abbreviations:** 5-ASA, 5-aminosalicylic acid; AUC, Area under the curve; CI, Confidence interval; NLR, Neutrophil-to-lymphocyte ratio; PLR, Platelet-to-lymphocyte ratio; SII, Systemic immune-inﬂammation index; NPR, Neutrophil-to-platelet ratio; PAR, Platelet-to-albumin ratio; CAR, C-reactive protein-to-albumin ratio; CLR, C-reactive protein-to-lymphocyte ratio. | | | | | |
